# Machine learning-driven electronic identifications of single pathogenic bacteria

**DOI:** 10.1038/s41598-020-72508-3

**Published:** 2020-09-23

**Authors:** Shota Hattori, Rintaro Sekido, Iat Wai Leong, Makusu Tsutsui, Akihide Arima, Masayoshi Tanaka, Kazumichi Yokota, Takashi Washio, Tomoji Kawai, Mina Okochi

**Affiliations:** 1 Department of Chemical Science and Engineering, School of Materials and Chemical Technology, Tokyo Institute of Technology, 2-12-1, O-okayama, Meguro-ku, Tokyo, 152-8552 Japan; 2 The Institute of Scientific and Industrial Research, Osaka University, Mihogaoka 8-1, Ibaraki, Osaka, 567-0047 Japan; 3 Department of Biomolecular Engineering, Graduate School of Engineering, Nagoya University, Furo-cho, Chikusa-ku, Nagoya, 464-8603 Japan; 4 National Institute of Advanced Industrial Science and Technology, Takamatsu, Kagawa 761-0395 Japan

**Keywords:** Biosensors, Microfluidics, Nanofabrication and nanopatterning, Nanopores

## Abstract

A rapid method for screening pathogens can revolutionize health care by enabling infection control through medication before symptom. Here we report on label-free single-cell identifications of clinically-important pathogenic bacteria by using a polymer-integrated low thickness-to-diameter aspect ratio pore and machine learning-driven resistive pulse analyses. A high-spatiotemporal resolution of this electrical sensor enabled to observe galvanotactic response intrinsic to the microbes during their translocation. We demonstrated discrimination of the cellular motility via signal pattern classifications in a high-dimensional feature space. As the detection-to-decision can be completed within milliseconds, the present technique may be used for real-time screening of pathogenic bacteria for environmental and medical applications.

## Introduction

Bacteria constitute a major class of pathogens in environments including water and food^1–3^. They are evolved in distinct ways to spontaneously move by using motile filamentous organelle protruding from the micrometer-scale bodies for invading in organisms and multiply themselves. While many of them are innocuous, there are also toxic strains that may bring detrimental impacts on health of individuals to global economy through causing infection disease outbreaks. Plate counting and polymerase chain reaction-based genomic analyses have thus been used as effective means to inspect presence or absence of bacteria in biosamples. However, the methods in general involve many pretreatment steps and thus time-consuming often requiring hours for the tests to be completed^4,5^, despite that the risk of infection outspread rises quickly in general as the microbes grow in number in the hosts. It is therefore of urgent importance to construct a reliable and highly-sensitive sensor for rapid analyses of viable microbes at an early stage of infection.

Here we report on use of solid-state micropores for label-free single-cell discriminations of five clinically-important pathogenic bacteria, *Staphylococcus aureus*, *Pseudomonas fluorescens*, *Salmonella enterica*, *Escherichia coli* and *Bacillus cereus*^6–9^, by their unique motility. The working mechanism is based on single-cell detections via Coulter principle that measures transient drops in the cross-membrane ionic current upon translocation of microbes through the conduit^10^. Unlike the conventional Coulter counters having relatively high thickness-to-diameter aspect ratio channel structures, a special care was taken in the present study to exploit it for detecting bacterial motility by employing a low aspect ratio pore architecture^11–13^ to make the ionic current more sensitive to multiple physical parameters of analytes such as shape^14,15^, surface charge density^16^, mass^17,18^, surface proteins^19,20^, and even the translocation motions^21^. Furthermore, since the enhanced sensor sensitivity is expected to yield more complicated ionic current signal patterns, we employed machine learning to pattern-analyze bacteria-derived fine features in the electrical signatures so as to compare and discern the multitude of physical properties of individual microbes in a high dimensional feature space.

## Results

**Figure 1:**
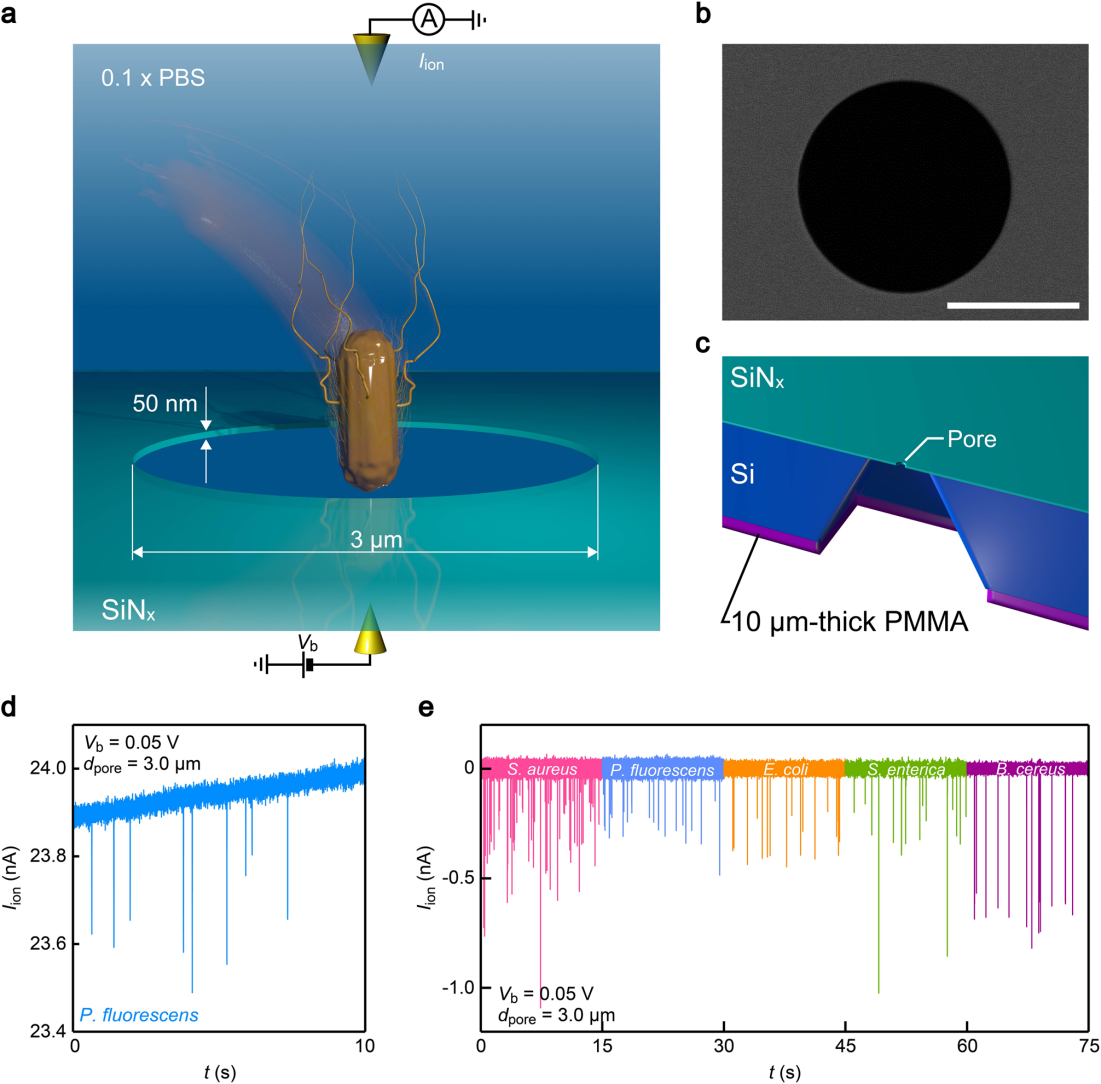
Single-bacteria detections using a low thickness-to-diameter aspect ratio micropore. (**a**) A sketch depicting a bacterium captured into a 3 μm-sized pore sculpted in a 50 nm-thick SiN_x_ membrane. The associated transient change in the ionic current *I*_ion_ through the pore was measured using a pair of Ag/AgCl electrodes under the applied voltage *V*_b_. (**b**) A scanning electron micrograph of a micropore. Scale bar denotes 2 μm. (**c**) A model depicting the cross-sectional view of the pore chip. On the backside of the substrate, 10 μm-thick polymethyl methacrylate (PMMA) layer was coated for reducing the device capacitance. (**d**) *I*_ion_ versus time (*t*) trace recorded in 0.1 × PBS containing *P. fluorescens* with a micropore of diameter *d*_pore_ = 3.0 μm under *V*_b_ = 0.05 V. (**e**) Resistive pulses observed in dispersion solution of the five pathogenic bacteria. Color coding is: pink = *S. aureus*, cyan: *P. fluorescens*, orange = *E. coli*, green: *S. enterica*, and purple = *B. cereus*. Open pore current is offset to zero.

The micropore consists of a lithographically-defined hole of diameter *d*_pore_ formed in a 50 nm thick SiN_x_ membrane (Fig. 1a,b). In experiments, bacteria dispersed in phosphate buffer saline (PBS) was added to one side of the membrane while the other side was filled with only PBS. Two Ag/AgCl rods were then used as electrodes to measure the ionic current *I*_ion_ flowing through the microscale conduit under the applied dc voltage *V*_b_. Backside of the substrate was coated with a micrometer-thick PMMA layer (Fig. 1c) to reduce the device capacitance for lowering high-frequency current noise^22^ as well as to ensure fast temporal resolution of the sensor^23^ for detecting rapid change in *I*_ion_ during the single-cell translocation.

The ionic current through the SiN_x_ pore of diameter *d*_pore_ = 3 μm was measured to be around 24 nA at *V*_b_ = 0.05 V (Fig. 1d). This manifests the predominant influence of the access resistance^24^ *R*_acc_ = *ρ*/*d*_pore_ = 2 MΩ on *I*_ion_ compared to the component *R*_pore_ = 4*ρL*/*πd*_pore_^2^ = 42 kΩ inside the channel, where *L* = 50 nm is the channel depth and *ρ* = 6 Ωm is the resistivity of the electrolyte buffer. Meanwhile, each time when a bacterium passed through the pore, it temporarily blocked the ion flow thereby caused a pulse-like *I*_ion_ drop (Fig. 1e).

**Figure 2:**
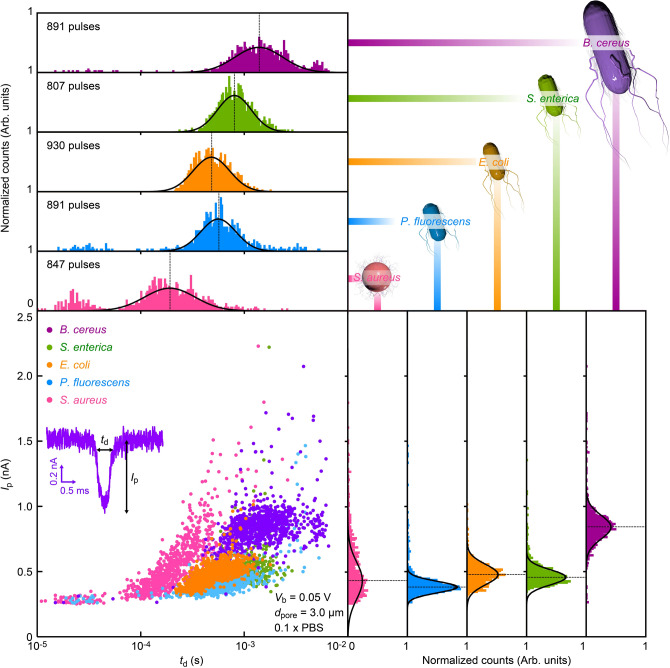
Resistive pulse patterns of pathogenic bacteria. The pulse height *I*_p_ versus width *t*_d_ scatter plots (bottom left) and corresponding histograms. The counts are normalized by the number of resistive pulses. Color code denotes: pink = *S. aureus*, cyan = *P. fluorescens*, orange = *E. coli*, green = *S. enterica*, and purple = *B. cereus* as also described schematically in the top right. Dotted lines point the center of *I*_p_ and *t*_d_ distributions defined by Gaussian fits.

The *I*_ion_ signatures are anticipated to reflect the intrinsic physical properties of the bacteria. To see if there is any difference in the resistive pulse profiles among the five species, therefore, we constructed scatter plots of the signal height *I*_p_ as a function of the width *t*_d_ that are known to represent the characteristic size and the time required for the cell to pass through the micropore, respectively (Fig. 2). As a result, we found significant overlap in their distributions, naturally interpreted as a consequence of their resembling micrometer-scale morphologies as confirmed by electron microscope observations (Table 1).

**Table 1:**
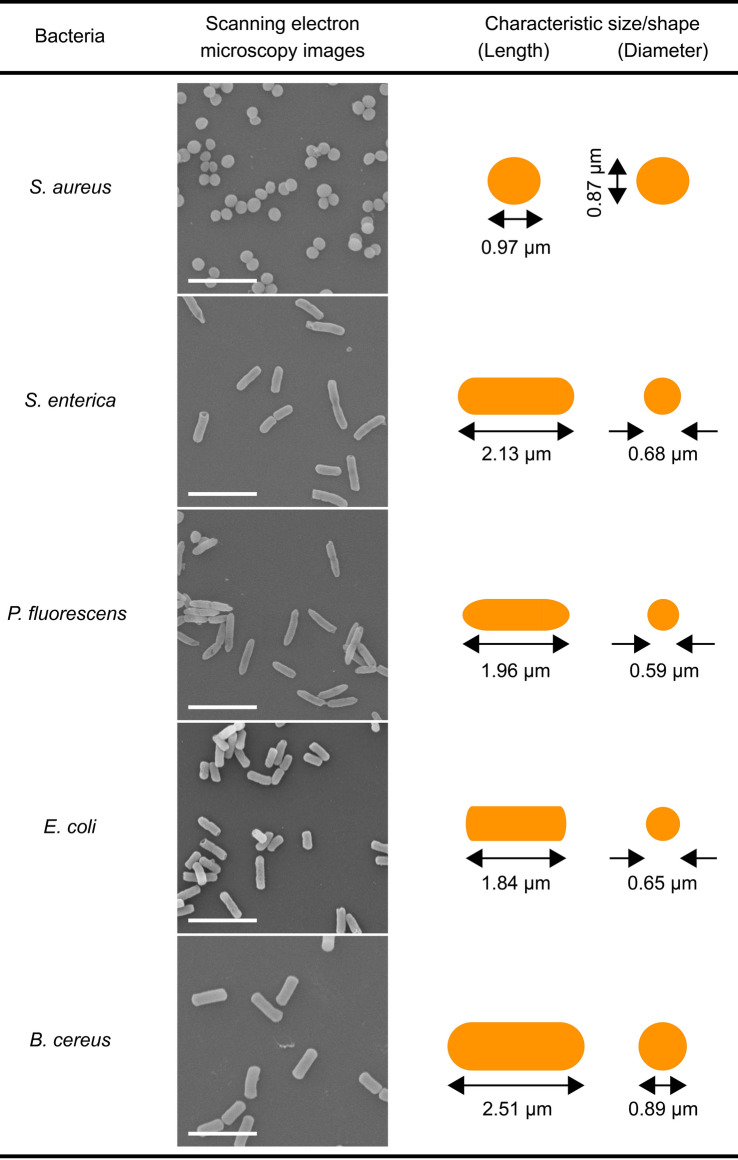
Size and shape of pathogenic bacteria.

Scale bars denote 5 μm.

**Figure 3:**
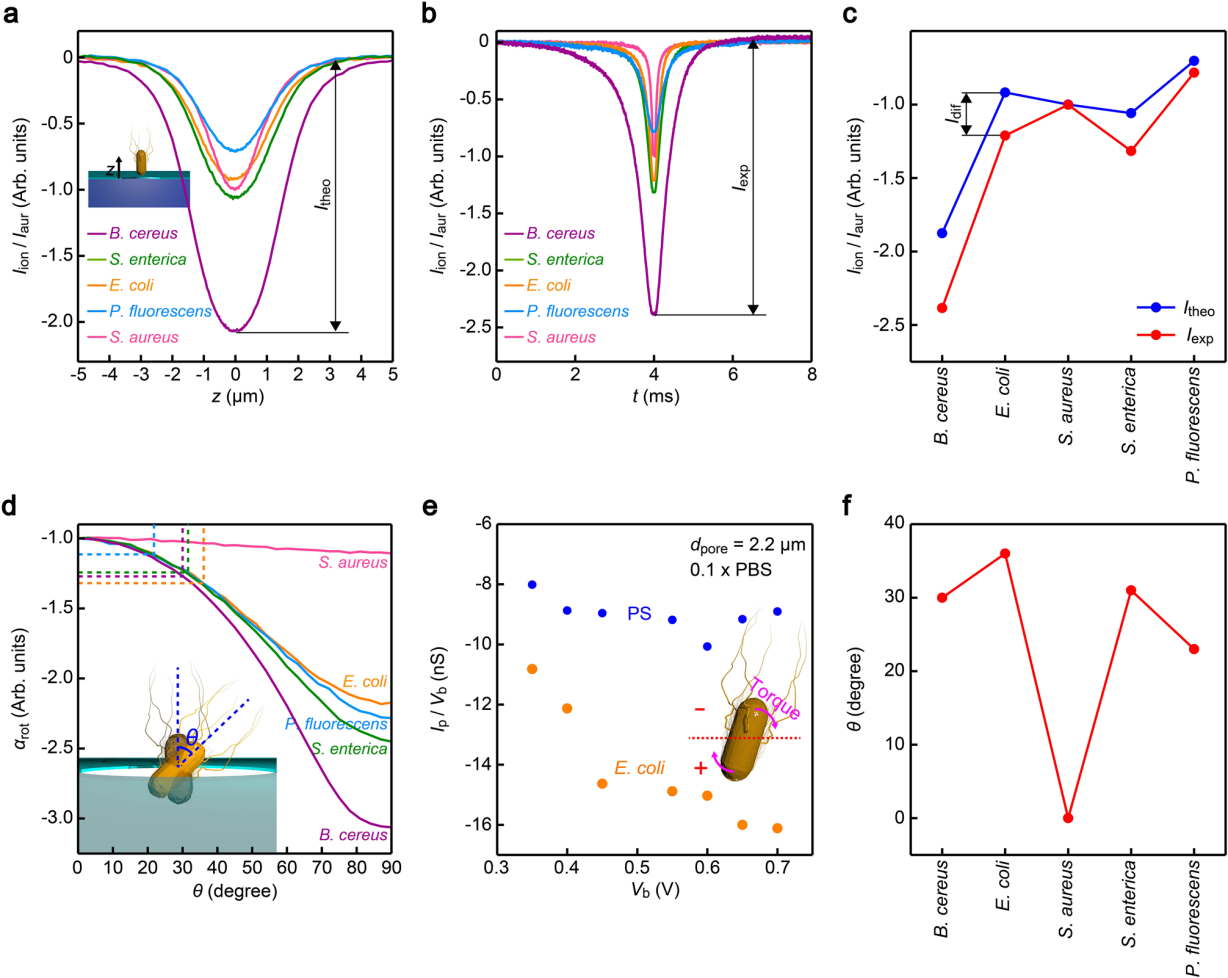
Bacterial orientation dependent resistive pulse waveforms. (**a**) Resistive pulses of the five bacteria simulated by a finite element analysis using COMSOL with a rod-shaped cell body moved through the 3 μm-sized pore in the 50 nm-thick SiN_x_ membrane along the axial direction. *z* scales the position of the center of mass of each bacterium from the middle of the channel as shown in the inset. Open pore current is offset to zero. The ionic current is normalized by the pulse height of *S. aureus* |*I*_aur_|. *I*_theo_ denotes the height of each pulse. (**b**) Average resistive pulses calculated from more than 800 resistive pulses obtained for each bacterium. The longitudinal axis is normalized by *I*_aur_. The heights of the pulses are described by *I*_exp_. (**c**) Plots of *I*_theo_ and *I*_exp_ for the five bacteria. *I*_dif_ represents the deviations between the simulation and the experimental data. (**d**) Theoretically-estimated change in the resistive pulse height by the rotation of the bacteria in the center of the micropore at the angle *θ*. *α*_rot_ represents the amount of increase in the heights with respect to those at *θ* = 0*.* Dashed lines point to *α*_rot_ deduced from (c) under an assumption of *α*_rot_ = *I*_diff_. (**e**) Voltage dependence of the resistive pulse heights of 1.1 μm-sized carboxylated polystyrene (PS) particles (blue) and *E. coli* (orange). Inset image explains a possible torque exerted on the bacteria upon moving through the micropore via the involved galvanotactic response against the electric stimuli. (**f**) *θ* back-calculated from *α*_rot_.

Under this circumstance, it becomes important to explore other features in the *I*_ion_ signatures that can better distinguish the pathogenic bacteria. For this, we first investigated the physical origins of the variations in the resistive pulse patterns by performing finite element simulations^13^ of the cross-membrane ionic current. We built three-dimensional models of the bacteria deduced from their morphologies depicted in the scanning electron micrographs. By changing the position *z* of the modeled structure along the axial direction, we obtained *I*_ion_ blockade characteristics for the five microbes (Fig. 3a), where we tentatively assumed an up-right orientation of the cell with respect to the membrane surface. The theoretical resistive pulses were then compared to the *I*_ion_ signals recorded in the experiments (Fig. 3b, see also Fig. S1). Here, we normalized the ionic current by the signal height for the quasi-spherical *S. aureus I*_aur_. The results revealed underestimations of the resistive pulse heights in the finite element analysis for the non-spherical microbes (Fig. 3c). This can be ascribed to tilted bacterial conformations during the actual translocation in the experiments that differ from the vertical configurations assumed in the simulations. Indeed, the model calculations revealed a non-linear increase in the pulse height with the bacterial tilting angle *θ* for all of the five species (Fig. 3d), whose effect is also found to be dependent on the microbial aspect-ratio motifs demonstrating a factor (*α*_rot_) of 1.1 for the quasi-spherical *S. aureus* while larger than 2 in cases of the other rod-shaped bacteria.

In this regard, it is noticeable that bacterial motility known as galvanotaxis would induce tilting motions under the focused in-pore electric field, wherein beating kinetics of cilia generates torque to rotate the rod-like cell body in response to the electrical stimulli^25,26^. To verify its possible role on the translocation dynamics, we extended the resistive pulse measurements to different *V*_b_ conditions (Fig. 3e, see also Figs. S2-S3). A control test (Fig. S4) on 1.1 μm-sized carboxylated polystyrene microbeads showed almost constant *G*_p_ = *I*_p_/*V*_b_ suggesting linear increase in the ionic current with the applied voltage following the ohm’s law. In sharp contrast, *E. coli* yielded a steady increase in *G*_p_ with *V*_b_. The result implies a higher chance of having more tilted rod-shaped body in the pore under the larger electric field via the galvanotaxis-derived faster rotation motions (Fig. 3e).

Taking the electric field-derived ciliary motions of microbes into account, we deduced their most-likely tilting angle in the micropore. When assuming that *I*_dif_ stems solely from the varying orientations of the cell bodies (Fig. 3c), the ratio *I*_exp_/*I*_theo_ can be regarded as equal to the deviation *α*_rot_ (Fig. 3e). From this, we roughly estimated *θ* that indicated prominent roles of the unique motility to render bacteria-specific conformations with *E. coli* being the most oblique one (Fig. 3f).

**Figure 4:**
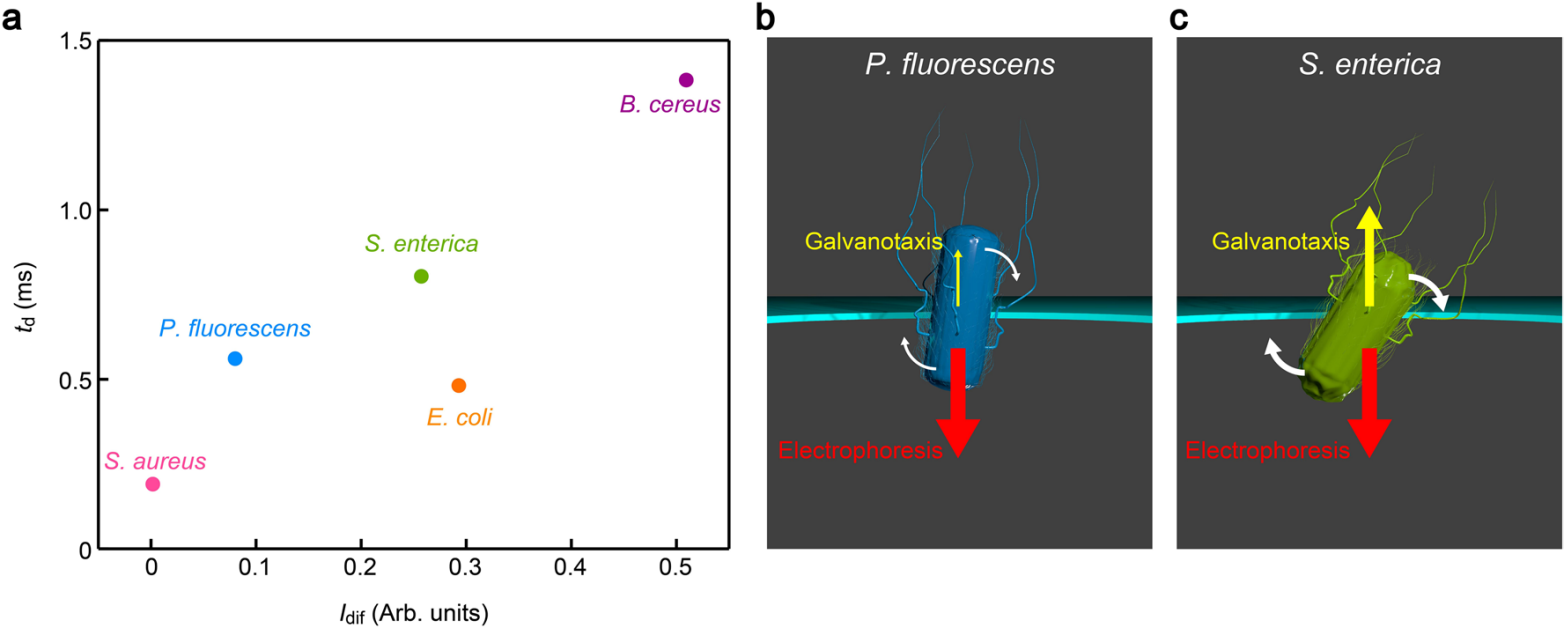
Galvanotaxis-derived retarded bacterial translocation. (**a**) Plots of the resistive pulse width *t*_d_ as a function of *I*_dif_. (**b**, **c**) *P. fluorescens* showing relatively small *I*_dif_ is predicted to exhibit weak galvanotactic response that leads to shorter *t*_d_ (**b**), while the translocation speed of *S. enterica* with larger *I*_dif_ is lowered more significantly by the field-induced bacterial motility (**c**).

Besides the rotation, the galvanotactic response is known to add a momentum to move the negatively-charged bacterium in direction opposite to the electrophoresis^25,26^. Indeed, plots of the resistive pulse width *t*_d_ against *I*_dif_ elucidated longer translocation time for the microbes exhibiting larger contributions of galvanotaxis (Fig. 4a), which can be interpreted as a consequence of the slower electrophoretic speed under the stronger back-force (Fig. 4b,c). *E. coli* is an exception displaying relatively short *t*_d_ that would be due to the shorter effective distance the large-*θ* bacterium travels within *t*_d_. The overall findings consistently indicated the prominent role of galvanotaxis on the *I*_ion_ signal profiles.

**Figure 5:**
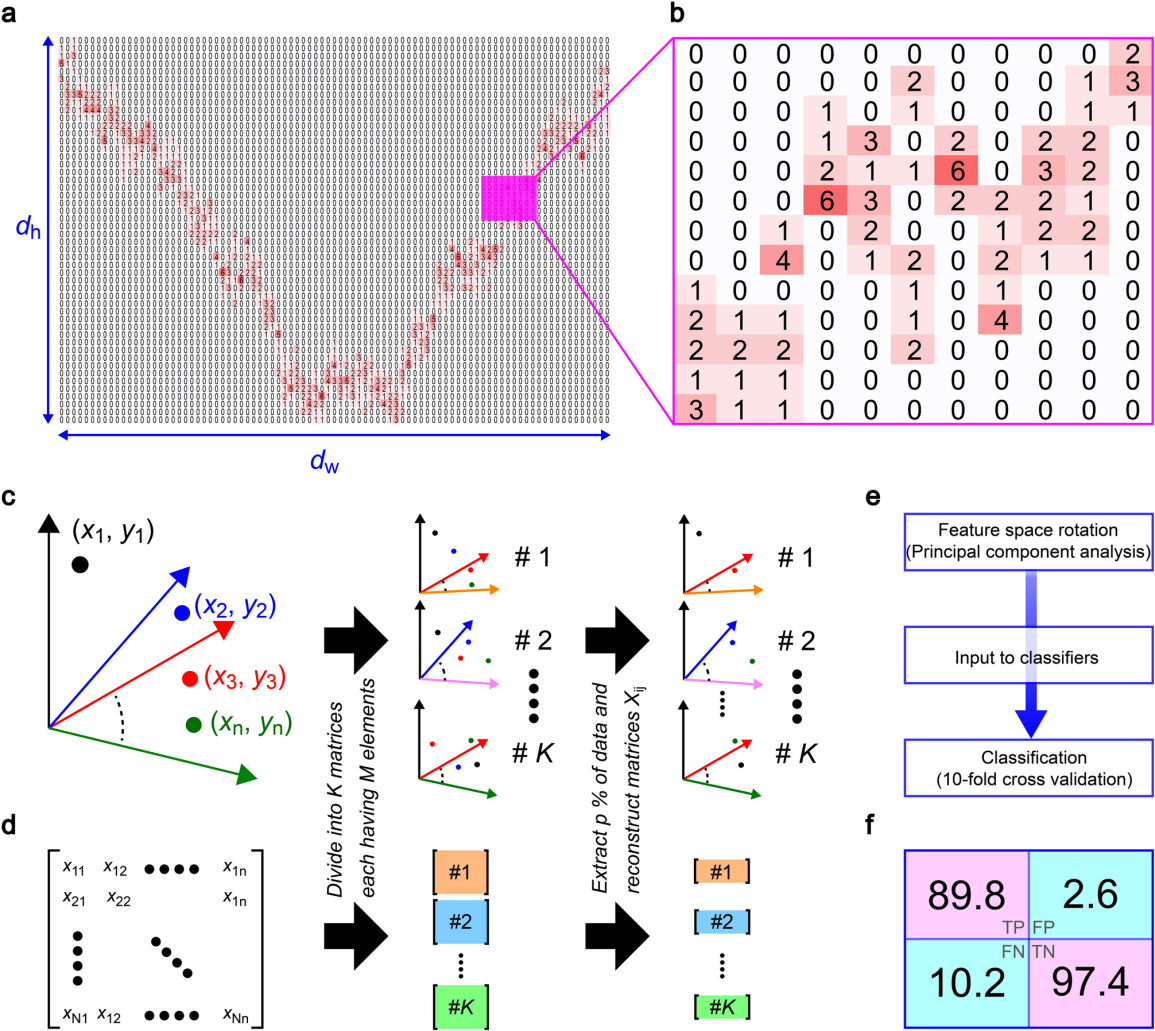
Machine learning-driven resistive pulse analyses. (**a**) Main feature parameters defined in the two-dimensional map of a resistive pulse in the *I*_ion_ – *t* domain. (**b**) The number of data points in each mesh of the *d*_h_ x *d*_w_ sections is used as feature parameters. (**c**, **d**) Schematic (c) and mathematic (d) explanations of the ensemble learning approach for classifying the resistive pulses in a high-dimensional feature space. (**e**) The algorithm includes a principal component analysis to select the effective features and redefine a new set of vectors accordingly for the classification. (**f**) Confusion matrix showing a high precision better than 89% to identify single-bacteria among the 5 different species. TP, TN, FP, and FN denote true positive, true negative, false positive, and false negative, respectively.

We now quantify the discriminability of the five bacteria by the resistive pulse shapes. Usually, the single-cell analysis is implemented by looking at the signal heights^10,27^ that allows statistical discriminations of bacterial species as far as there is clear difference in the cell size^28–31^. On the other hand, the above results elucidated the peculiar sensitivity of the ionic current blockage in the low-aspect-ratio micropores on non-volume properties including the bacterial shape and motility. This calls for an analytical method that enables to leverage not only the pulse height but also finer features for distinguishing the ionic current signals. Therefore, we herein employed a classifier ensemble approach of Rotation Forest^32^ in WEKA^33^ to interrogate the resistive pulse patterns in a high-dimensional feature space. Specifically, we meshed the *I*_ion_—time domain into *d*_h_ × *d*_w_ regions (Fig. 5a) and counted the number of plots in each section (Fig. 5b). We also extracted researcher-crafted parameters from the signals such as the pulse height *I*_p_, width *t*_d_, bluntness *β*, onset angle *θ*_on_, area *A*, pulse asymmetry *r*_m_, and inertia *I*_lat_ and *I*_ver_ as summarized in Fig. S5. All of these were randomly selected to create 60 feature vectors and utilized for training 67 Rotation Forest ensembles (Fig. 5c,d). The algorithm then carried out the first classification for the resistive pulse data sets of known bacteria. Afterward, it internally exhibited a principal component analysis to choose the effective parameters with respect to the pretest scores, *i.e.* the number of correct outputs for judging labelled input signals, and subsequently used them to newly generate feature vectors for the post single-pulse discriminations (Fig. 5e). In this way, one can simultaneously use the multiple features on the same footing for classifying the varying pulse patterns reflecting the stochastic translocation motions of the motile bacteria.

The actual analysis was exhibited for randomly-chosen 87 resistive pulses. Among the signals, 79 were used for training classifiers to testify the discriminability of the bacteria by actually implementing resistive pulse classification for the rest of 8 pulses (tenfold cross validation). From the output of the algorithm on each labelled signals, we estimated the rate of true-negative, true-positive, false-negative, and false-positive cases. The result is summarized in a form of confusion matrix (Fig. 5f) that illustrates the high discriminability of the five bacteria with *F*-measure score *F*_meas_ reaching 0.91, which suggests 91% accuracy for identifying the bacteria from the *I*_ion_ signature profiles at a single-cell level. This discriminability can be judged as good considering the worst case be *F*_meas_ = 0.2 by a random guess of the five species.

It is of interest to know what enabled the single-bacteria discriminations. Nonetheless, it is not possible to figure out which of the researcher-defined parameters played an important role on the classifications due to the non-physical meaning of the internally-created feature vectors in the ensemble learning algorithm. Yet, we could at least confirm that the results were not affected by factors other than physical features of the bacterial cells. For instance, the bacterial concentration in the suspension was found to cause no notable influence on the signal waveforms (Fig. S6). This is ascribed to the principle of the ionic current blockage that reflects only the physical properties of objects passing through the sensing zone. Moreover, dead cells were observed to be not able to transit the pore (Fig. S7), and hence they were not detected, due presumably to the denatured surface proteins with less charges that render inadequate electrophoretic forces to draw the cells captured into the channel. In fact, the present study used fresh bacterial samples by cultivating on the day of the resistive pulse measurements to minimize the population of dead cells. Although this does not rule out the possible translocation of cell fragments during the experiments, their small sizes are naturally anticipated to cause ionic current drops that are too small to be detected under the given noise floor (Fig. S8).

While the training process is laborious and time-consuming due in part to the involved pulse extractions from the *I*_ion_-*t* data recorded at the 1 MHz sampling rate, the actual single-pulse classification per se requires less than a millisecond with a standard CPU. The machine-learning-driven resistive pulse analysis can therefore be a real-time method for label-free identifications of single-bacteria. The sensor concept is also viable to virtually any bacteria as long as they can be put through the sensing zone, the capability of which may find various bioanalytical applications let alone the case sensitive pretreatments required to ensure no inclusion of oversized objects in samples that may potentially clog the channel.

## Conclusions

Single-cell identifications of clinically important pathogenic bacteria were examined by a machine learning-driven resistive pulse analysis using a low aspect-ratio micropore. The shallow channel structure rendered a particular sensitivity of the blockade ionic current to the three-dimensional translocation motions of the microbes that enabled to find distinct signatures of their unique motility under the imposed electric field in the signal profiles. In order to compare and discern the bacteria-specific nontrivial features in the resistive pulses, we exhibited data classification based on an ensemble-learning algorithm in a high-dimensional feature space, which was proven useful to detect and identify the physically resembling five bacteria in real time at the single-cell level.

## Methods

### Preparation of bacteria

Following bacteria were used as model microorganisms for micropore analysis. *Staphylococcus aureus* (No. 13276, NITE Biological Resource Center, Japan), *Pseudomonas fluorescens* (isolated strain from food sample donated from Meiji Co. Ltd., Japan), *Salmonella enterica* (JCM 1652, RIKEN BioResource Center, Japan), *Escherichia coli* (No. 3972, NITE Biological Resource Center, Japan) and *Bacillus cereus* (JCM 2152, RIKEN BioResource Center, Japan). *E. coli*, *B. cereus*, *S. aureus* were cultivated aerobically in Trypticase Soy Broth (pH 7.3) containing 1.7% Bacto tryptone, 0.3% Bacto Soytone, 0.25% Glucose, 0.5% Sodium chloride, 0.25% Dipotassium hydrogen phosphate in ultrapure water. *S. enterica* and *P. fluorescens* were cultivated aerobically in Nutrient Broth (pH 7.0) containing 0.5% Bacto peptone, 0.3% Difco Beef extract and 0.5% Sodium chloride in ultrapure water. *E. coli*, *S. aureus* and *S. enterica* were cultured at 37˚C while *B. cereus* and *P. fluorescens* were cultured at 30 °C for 4 h after preculture under same conditions. After culture, cells were centrifuged at 1000 g for 10 min, washed twice and resuspended in 0.1 × PBS, phosphate buffered saline containing 0.08% sodium chloride, 0.002% potassium chloride, 0.012% sodium monohydrogen phosphate, and 0.02% potassium dihydrogen phosphate at pH 7.4.

### Fabrication of low-aspect-ratio micropores

A Si wafer diced into 30 mm square chips was used as a substrate. On the both sides of the Si layers, there were 50 nm thick SiN_x_ formed by low-pressure chemical vapor deposition. The bottom side of the SiN_x_ was partially removed by reactive ion etching through a metal mask having a square window of 1 mm × 1 mm size. The exposed Si was immersed in KOH aq. and heated to 80 degrees Celsius for wet etching, which led to formation of a SiN_x_ membrane at the other side of the chip. On the membrane, electron beam resist was spin-coated and baked at 180 degrees Celsius. A circle of 3 μm diameter was delineated by electron beam lithography. After development, the residual resist was used as a mask to open the micropore by isotropic reactive ion etching of the exposed SiN_x_.

### Back-side polymer coating

On the back-side of the micropore chip, we spin-coated a polymethyl methacrylate (PMMA) layer of about 5 μm thickness. After baking, we irradiated electron beam from the front side of the surface at 50 μm around the micropore followed by development to remove the PMMA in the micropore.

### Micropore sealing

Two blocks made of polydimethylsiloxane (PDMS) were used to seal the micropore. These blocks were made by curing PDMS on a Si substrate with an SU-8 layer patterned to have a straight motif of 15 mm length and 100 μm thickness. After polymerization in oven at 80 degrees Celsius, the blocks were cut out with knife. Because of the SU-8 pattern, there was a fluidic channel formed on the bottom surface of the PDMS block. In prior to the sealing, the channel side of the PDMS block as well as the micropore chip was exposed to oxygen plasma for surface activation. Subsequently, the two surfaces were attached together for eternal bonding. This process was repeated again for another PDMS block to seal the other side of the micropore.

### Ionic current measurements

Through holes punched in the PDMS block, we injected PBS containing bacteria at one side of the micropore and bacteria-free buffer at the other side. Ag/AgCl rods were then placed at the both sides of the micropore. The ionic current through the micropore was measured by applying dc voltage between the Ag/AgCl and recording the output current through a custom-built preamplifier backed by a fast digitizer at a 1 MHz sampling rate.

### Finite element analysis

The ionic current through the micropore was simulated by a finite element method. A three-dimensional model was constructed, which was composed of a cylindrical cell of 50 μm diameter and 100 μm height with a 50 nm thick SiN_x_ disk at the middle. A 3 μm-sized micropore was opened in the disk and the cell was filled with water containing Na^+^ and Cl^-^ ions at a concentration of 13.7 mM. The electric potential at one end was set to 0 V while the other side to + 0.05 V. The ionic current was calculated by solving Poisson-Nernst-Plank and Navier–Stokes equations in a self-consistent manner using COMSOL Multiphysics 5.4.

### Machine learning-driven resistive pulse pattern analysis

Classification of resistive pulse patterns was performed using the machine learning workbench WEKA with Rotation Forest ensembles each employing a distinct base classifier. Researcher-crafted feature parameters were defined and exploited in a machine learning-based classification of resistive pulses. These are: the height *I*_p_; the width *t*_d_; the bluntness at the pulse apex *β*_apex_; the onset angle *θ*, the pulse peak position *r*; the area *A*; the ratio *r*_m_ between the area before and after the peak maximum; the inertia *I*_m_ and *I*_w_ calculated with respect to longitudinal and transverse axes, respectively (detailed definitions of these parameters are explained in Fig. S5). The feature parameters were obtained from each resistive pulse data. Each parameter was coupled to the current vector and the time vector to create feature vectors, which were then used for training classifiers. 90% of the 87 resistive pulses of known bacteria were used for the training, and the trained classifiers judged the other 10%. The average accuracy of the bacterial discrimination was assessed by repeating this procedure ten times through interchangeably changing the teacher data and the test data (tenfold cross validation). The *F*-measure score *F*_meas_ = 2*P*_Pre_*P*_Rec_/(*P*_Pre_ + *P*_Rec_) was deduced as an average of the whole combinations of the classifiers and the feature vectors, where *P*_Pre_ and *P*_Rec_ are the precision and recall calculated through TP/(TP + FP) and TP/(TP + FN), respectively, with TP, FP, and FN being the number of true-positive, false-positive, false-negative cases, respectively, estimated from the output for the labelled resistive pulse data.

## Supplementary information

Supplementary Figures.

## Data Availability

All data and material are available for readers in methods and supplementary information.
