# Supplementary material for: Machine learning-driven electronic identifications of single pathogenic bacteria
Source: Sci Rep. 2020 Sep 23;10:15525. doi: 10.1038/s41598-020-72508-3 (PMC7512020; doi:10.1038/s41598-020-72508-3)
Supplement: Supplementary file 1 — Supplementary Figures. [file 41598_2020_72508_MOESM1_ESM.pdf]

Supplementary Information

Machine learning-driven electronic identifications of single pathogenic bacteria

Shota Hattori, Rintaro Sekido, Iat Wai Leong, Makusu Tsutsui, Akihide Arima, Masayoshi Tanaka, Kazumichi Yokota, Takashi Washio, Tomoji Kawai, and Mina Okochi

E mail: tsutsui@sanken.osaka-u.ac.jp, washio@ar.sanken.osaka-u.ac.jp, kawai@sanken.osaka-u.ac.jp, or okochi.m.aa@m.titech.ac.jp

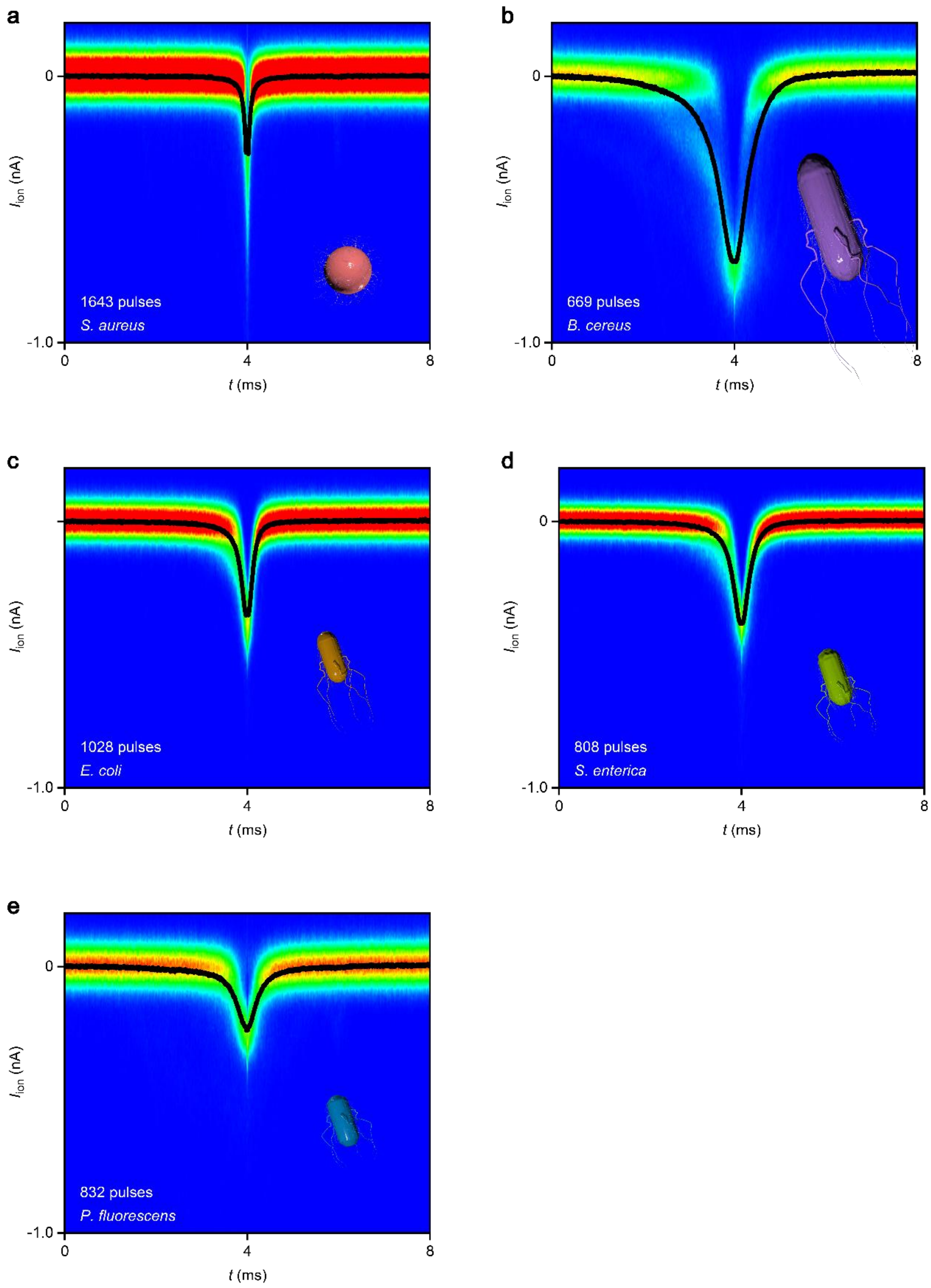

Figure S1. a-e, Resistive pulses displayed in forms of two-dimensional histograms for *S. aureus* (a), *B. cereus* (b), *E. coli* (c), *S. enterica* (d), and *P. fluorescens* (e). Black plots are the average of more than 600 pulses measured.

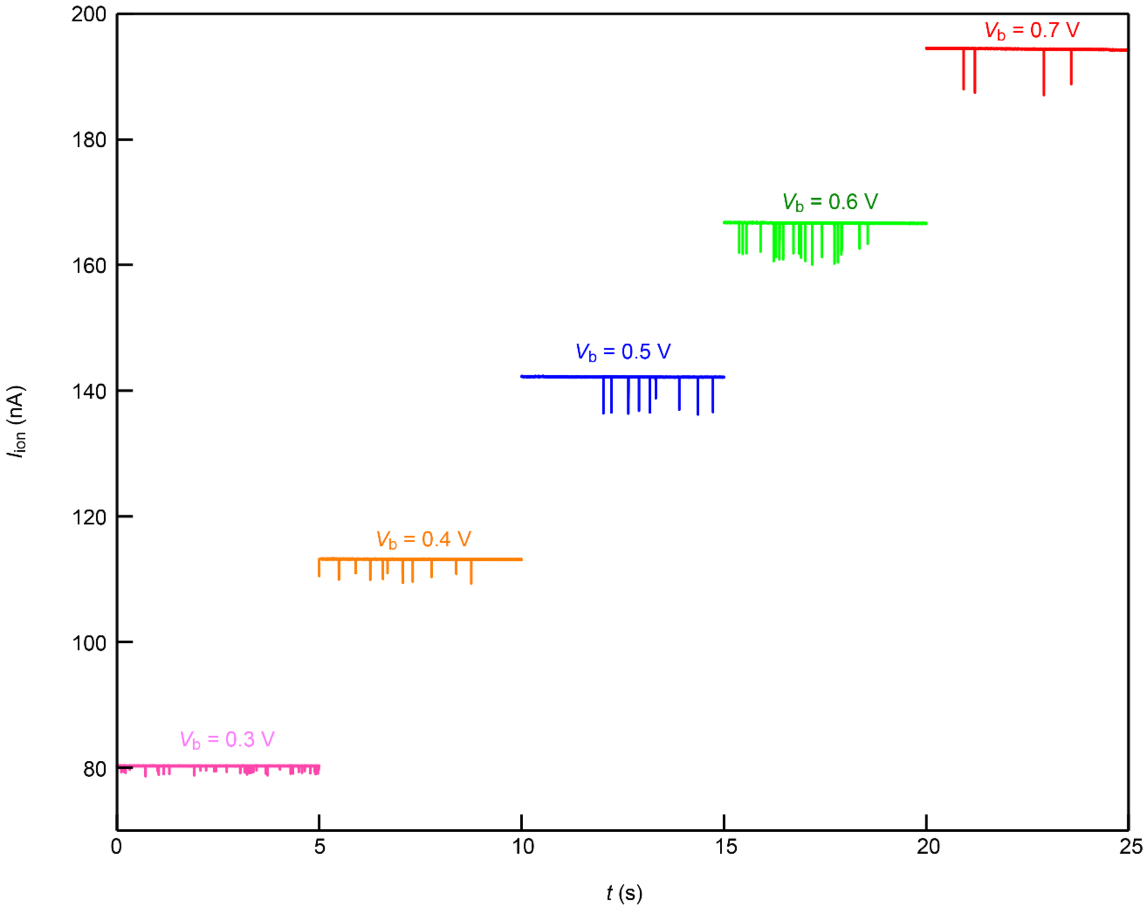

Figure S2. Ionic current traces recorded in dispersion solution of *E. coli* in 0.1 x PBS with 2.2 μm-sized pore under various cross-membrane voltage V_b form 0.3 V to 0.7 V. The open pore current increase almost linearly with V_b while the resistive pulse height shows non-linear increase suggestive of the galvanotactic response of the bacteria.

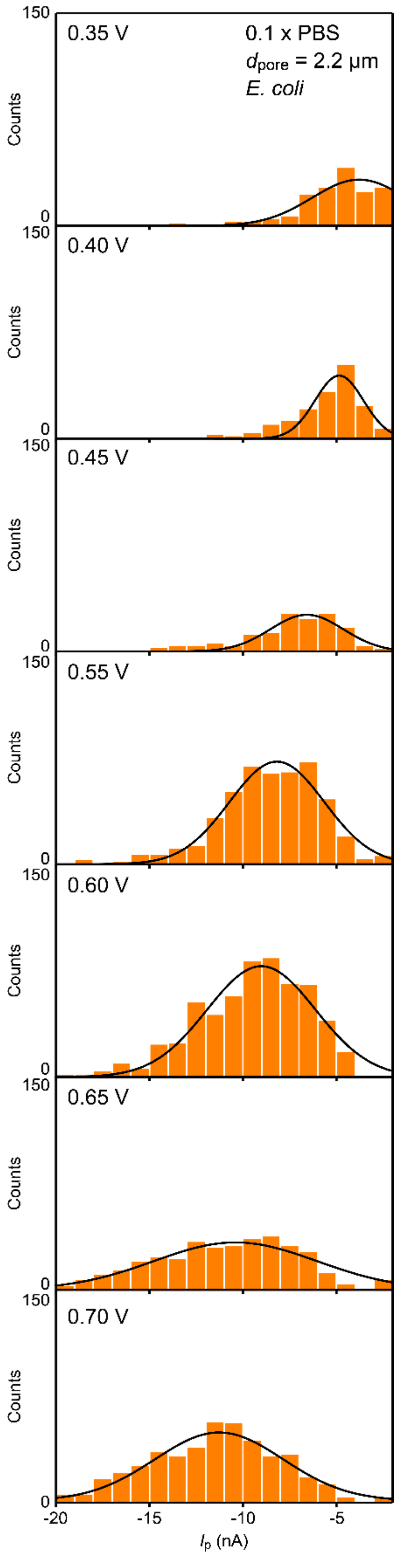

Figure S3. Voltage dependence of the resistive pulse height I_p for *E. coli* translocated through a 2.2 μm-sized pore in a 50 nm-thick SiN_x membrane. Black curves are Gaussian fits to the distributions.

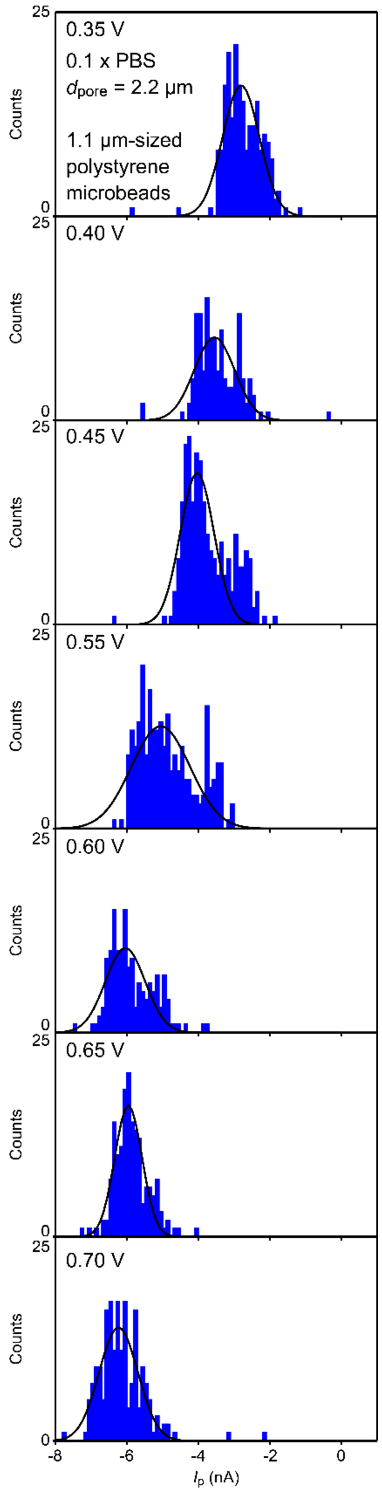

Figure S4. Voltage dependence of the resistive pulse height I_p for carboxylated polystyrene microbeads of 1.1 μm diameter translocated through a 2.2 μm-sized pore in a 50 nm-thick SiN_x membrane. Black curves are Gaussian fits to the distributions.

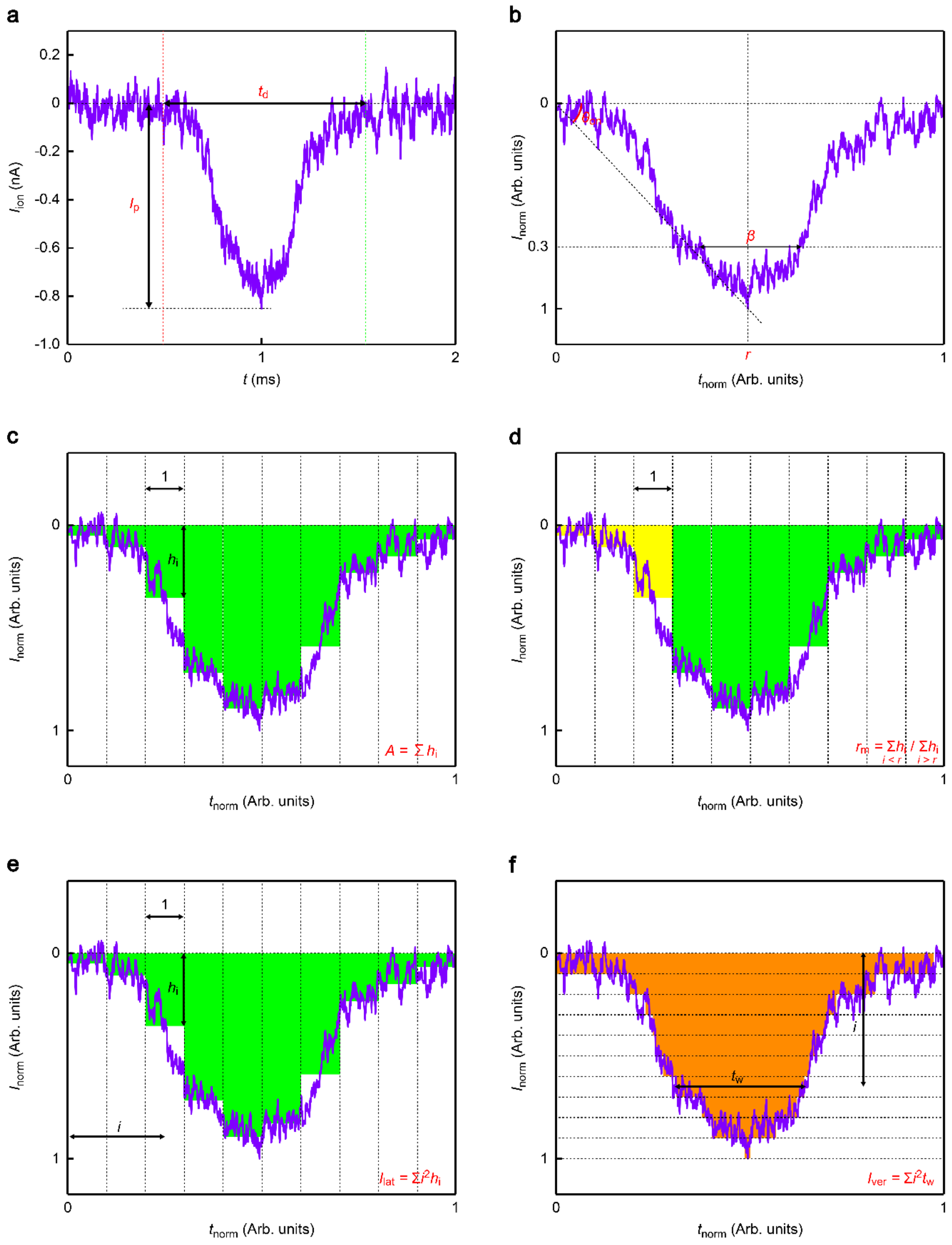

Figure S5. Researcher-crafted feature parameters. **a,** Resistive pulse height I_p and width t_d. Red and green dotted lines denote the start and the end points of the pulse signal defined as I_{ion} crossing 5σ. **b,** The angle θ_{on} and the pulse

position r extracted from the normalized resistive pulses. The bluntness β describing the width at 30 % from the pulse top. Here, the normalization was conducted by using the starting and end points of the resistive pulses for the time (t_{norm}) while dividing I_{ion} by I_p for the ionic current. **c,** The area A taking summation of the height h_i denoting the average height of the n-divided sections. **d,** The ratio r_m between the area at the pulse onset (yellow) and tail (green). **e-f,** The inertia I_{lat} (e) and I_{ver} (f) with respect to the lateral and vertical axes, respectively.

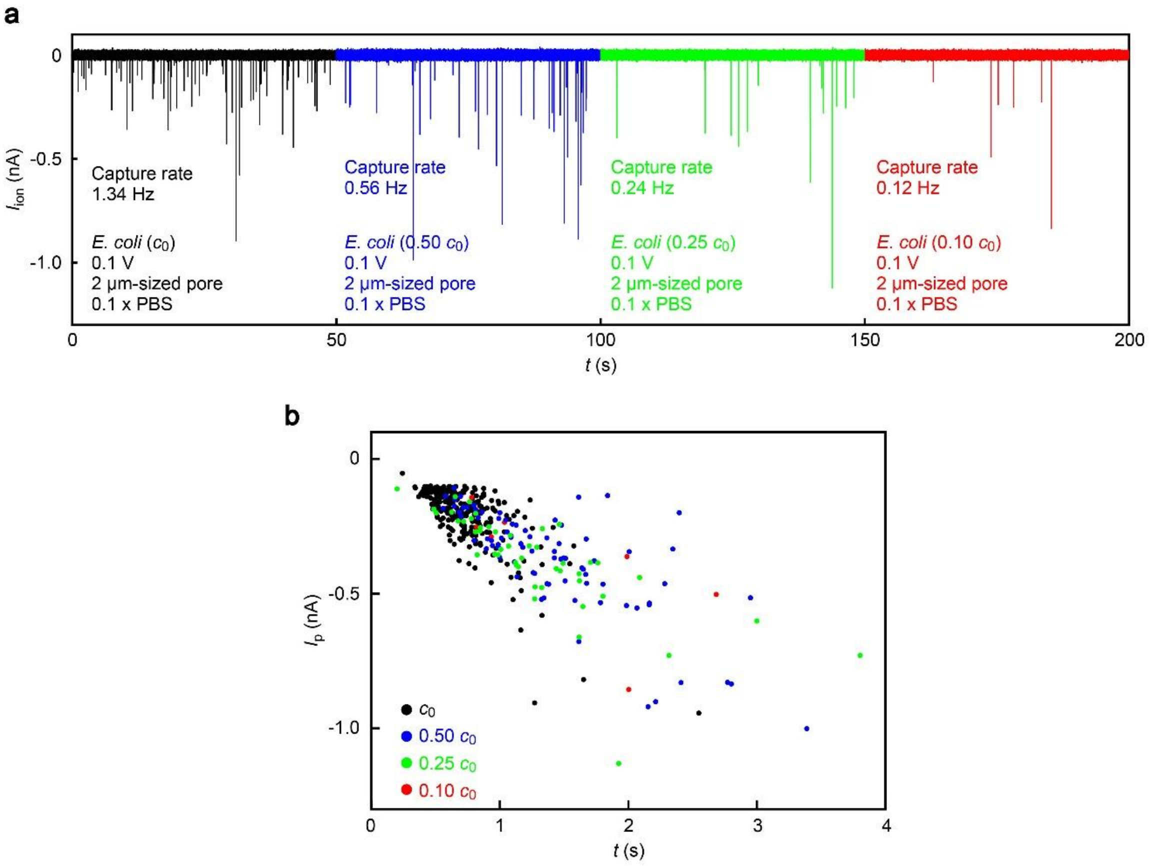

Figure S6. Dependence of resistive pulse height and width on bacterial concentrations. **a,** Ionic current traces recorded using a 2 μm-sized micropore in a 50 nm-thick SiN_x membrane in 0.1 x PBS containing *E. coli* at various concentrations: black = c_0, blue = 0.50 c_0, green = 0.25 c_0, red = 0.10 c_0. The occurrence rates of the pulse signals decrease with decreasing the bacterial concentration. **b,** Resistive pulse height I_p and width t_d under different bacterial concentrations. There is no notable difference in the scatter plots manifesting that the bacterial concentration affects only the capture rates.

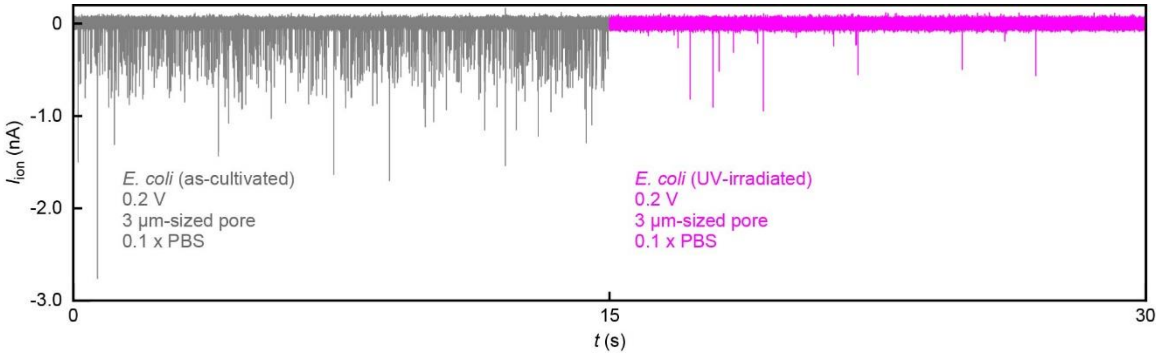

Figure S7. Influence of dead cells on the ionic current traces. The ionic current through a 3 μm-sized micropore in a 50 nm-thick SiN_x membrane showed numerous resistive pulses in a suspension of as-cultivated *E. coli* (grey). After irradiating ultraviolet (UV) light (20 mW/cm^2) on the same solution for 15 minutes (pink), the signal rate decreased appreciably while no obvious change in the pulse height indicating. The results indicate that dead cells cannot pass through the pore due to the less amount of negative charges on the cell surface for the electrophoretic translocation.

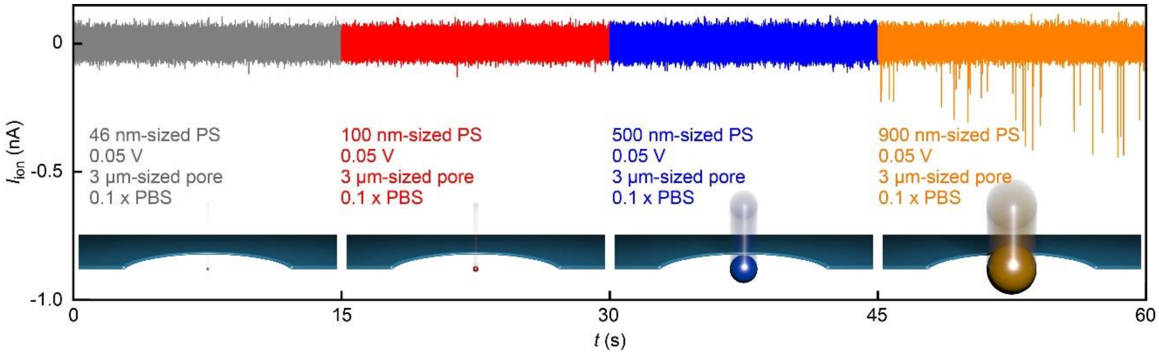

Figure S8. Sensitivity of ionic current on particle size. The ionic current through a 3 µm-sized micropore in a 50 nm-thick SiN_x membrane recorded in 0.1 x PBS containing carboxylated polystyrene (PS) nanoparticles of various diameter d_{ps}: grey = 46 nm, red = 100 nm, blue = 500 nm, orange = 900 nm. Insets depict the size of nanoparticles translocating through the micropore. Particles of size less than 900 nm caused ionic blockade current that was too weak to be detected under the given noise floor.
